# Biological Activities of Aqueous and Organic Extracts from Tropical Marine Sponges

**DOI:** 10.3390/md8051550

**Published:** 2010-04-28

**Authors:** Kristina Sepčić, Silke Kauferstein, Dietrich Mebs, Tom Turk

**Affiliations:** 1 Department of Biology, Biotechnical Faculty, University of Ljubljana, Večna pot 111, 1000 Ljubljana, Slovenia; 2 Institute of Legal Medicine, University of Frankfurt, Kennedyallee 104, 60596 Frankfurt, Germany

**Keywords:** tropical marine sponges, hemolysis, hemagglutination, antibacterial activity, acetylcholinesterase (AChE) inhibition, protein phosphatase 1 (PP1) inhibition/activation

## Abstract

We report on screening tests of 66 extracts obtained from 35 marine sponge species from the Caribbean Sea (Curaçao) and from eight species from the Great Barrier Reef (Lizard Island). Extracts were prepared in aqueous and organic solvents and were tested for hemolytic, hemagglutinating, antibacterial and anti-acetylcholinesterase (AChE) activities, as well as their ability to inhibit or activate cell protein phosphatase 1 (PP1). The most interesting activities were obtained from extracts of *Ircinia felix*, *Pandaros acanthifolium*, *Topsentia ophiraphidites*, *Verongula* r*igida* and *Neofibularia nolitangere*. Aqueous and organic extracts of *I. felix* and *V. rigida* showed strong antibacterial activity. *Topsentia* aqueous and some organic extracts were strongly hemolytic, as were all organic extracts from *I. felix*. The strongest hemolytic activity was observed in aqueous extracts from *P. acanthifolium*. Organic extracts of *N. nolitangere* and *I. felix* inhibited PP1. The aqueous extract from *Myrmekioderma styx* possessed the strongest hemagglutinating activity, whilst AChE inhibiting activity was found only in a few sponges and was generally weak, except in the methanolic extract of *T. ophiraphidites*.

## 1. Introduction

Marine sponges (Porifera) are primitive metazoans. Sponge species are predominantly marine and sessile. They produce a plethora of compounds that protect them from predators and/or possibility from being infected and fouled by other marine organisms [1]. Marine sponges are considered to be true “chemical factories” producing hundreds of unique chemical compounds, many of which have been isolated and their structure determined, but their biological roles and activities are still largely unknown [2].

Despite the popular and somewhat overexploited statement that marine sponges are an important source of new bioactive compounds that may be used in various biomedical applications, so far only a few have found such an application [3]. Nevertheless, a large number of sponge secondary metabolites show interesting biological activities, for example calyculins from *Discodermia calyx*) [4], discodermolide from *D. dissoluta* [5], latrunculins from *Latrunculia magnifica* [6–9], and spongistatins from *Spongia* sp. and *Spirastrella* sp. [10–12]. They are cytotoxic, inhibit cell proliferation and could be used as chemotherapeutics. These compounds differ structurally and act on different cytoskeletal elements, but have similar antiprolific and antitumoral activities.

Remarkable progress has been made with halichondrin B initially found in the Japanese marine sponge *Halichondria okadai* [13]; later, similar compounds were also found in other sponges such as *Phakelia carteri* [14] and *Lissodendoryx* sp. [15]. Halichondrin B, as a potential chemotherapeutic agent, has advanced to preclinical studies, however, due to the limited supply of the natural compound, a derivative of halichondrin B, E7389, was synthesized which proved to be a significantly more effective antitumor agent in animal models. As a result, E7389 was approved for clinical trials in 2001. Early results from a Phase II trial reported in December 2005 indicated that 15 percent of breast cancer patients in that trial, whose cancer was resistant to other treatments, responded to treatment with E7389. The National Cancer Institute is planning several Phase II clinical trials to test the effectiveness of E7389 in treating a variety of tumors including ovarian, prostate, bladder, pancreatic head and neck cancers [16]. Other examples of pharmaceuticals based on marine sponge natural products are Ara-A and Ara-C obtained from *Cryptotethya cripta* both of which are marketed as antiviral and anticancer drugs [3].

Despite the fact that cytotoxicity is the most widespread characteristic of compounds isolated from marine sponges, they also possess other activities. Due to the sessile nature of sponges it is not surprising that many of their natural products show strong antifouling activities. Several reviews on such compounds have been published recently [17,18].

So far only a few sponge metabolites have been reported to act as receptor/channel modulators or enzyme inhibitors, these include compounds from *Penares* sp. such as azetadins which inhibit protein kinase C [19,20] and penaramides that bind to the N-type calcium channels thus competing with ω-conotoxin binding sites [21], cyclostellettamines from *Stelletta maxima*, alkylpyridinium cyclic dimers that bind to muscarinic receptors of type M_1_, M_2_ and M_3_ [22], linear peptides from *Theonella swinhoei*, that act as inhibitors of thrombin [23] and serine proteases [24], and alkypyridinium polymers, isolated from *Reniera sarai*, which strongly inhibit AChE and may act as anticancer compounds affecting cholinergic system expressed in a variety of cancer cell lines [25–27]. A large number of secondary metabolites from a variety of marine sponges were reported to possess antiviral, antifungal and antibacterial activities, among them many bromotyrosine derivates [2].

In the present study we report the screening results of several biological activities found in aqueous and organic extracts from 43 tropical marine sponge species that could possibly lead to the discovery of novel compounds of pharmacological interest.

## 2. Results and Discussion

The majority of Caribbean and Australian tropical marine sponge extracts tested in this study possessed at least one biological activity (Suppl. Table 1a and 1b). However, a substantial amount of material was used in these preliminary assays, and therefore dilutions of the active samples were also tested. A small number of sponge extracts exhibited selective toxicity. After dilution of these extracts their selective toxicity became more evident. On the other hand many extracts exhibited broad toxicity which was almost completely lost after dilution. Broad toxicity could be attributed to the large number and high concentration of different compounds in the undiluted extracts. In the case of crude samples, we cannot overlook the fact that these compounds may act synergistically resulting in broad toxicity. Due to the different amounts of different compounds in the tested samples the level of certain compounds after dilution became to low to be effective, and only those whose concentrations were still high enough prevailed. As an example, undiluted organic extracts from *Agelas clathrodes* (#81) were strongly hemolytic and showed substantial AChE inhibitory activity. After dilution both activities were almost completly lost (Tables 1 and 3). Extracts from *Ircinia felix* (#59), on the other hand showed strong hemolytic and considerable AChE inhibitory activity. After dilution the former activity was completely preserved while the later was completely lost. Undiluted organic extracts from the same sponge also showed moderate PP1 inhibition, while heated aqueous extracts exhibited modest activation of the same enzyme. After dilution the former activity was lost while the later remained unchanged (Tables 1 and 4).

Hemolytic activity was present in only a few aqueous samples, whereas the same activity was observed in nearly half of the organic extracts. Acetone extracts possessed the highest levels of hemolytic activity, followed by butanol and methanol extracts; the latter contained the highest amount of extracted material. Aqueous extracts whose hemolytic activity was lost after heating were of special interest as this indicated the presence of a proteinaceous active compound(s). Based on these criteria such hemolytic proteins may be present in *Pandaros acanthifolium* (#76), however a second specimen from a different location (#14) did not exhibit any hemolytic activity. The unheated aqueous extract from *Spheciospongia vesparium* (#69) was hemolytic, but again another specimen (#45) from a different location was not. These results indicate that the same species from a different location may harbor different chemistry possibly originating from endosymbiotic organisms present in the particular collected specimen. Such observations were quite common in this study. Another interesting species that showed strong hemolytic activity was *Topsentia ophiraphidites* (#99). In this case, the active compound responsible for hemolytic activity was ascribed to be non-proteinaceous in nature, as its activity was retained after heating. Hemolytic assays using diluted samples of aqueous sponge extracts confirmed that *P. acanthifolium*, *S. vesparium* and *T. ophiraphidites* contained the highest levels of hemolytic activity. The most active organic extracts after dilution were those from *Agelas clathrodes* (#81), *Ircinia felix* (#59) and *Lissodendoryx colombiensis* (#110). All three extracts from *I. felix* possessed considerable hemolytic activity. Of the two other *Ircinia* species only one organic extract was active (Table 1). Hemolytic proteins isolated from marine sponges are rare. Two such examples are suberitin, isolated from the Mediterranean sponge *Suberites domuncula* [30] and a hemolysin from *Tethya lincurum* [31]. To date no hemolytic compounds have been reported from the marine sponges used in this study. An aqueous extract from *S. vesparium* was previously reported to exhibit low toxicity in mice, however this toxicity was not attributed to hemolytic activity [32].

Moderate hemagglutinating activity has been detected in 14 aqueous sponge extracts, one showed activity in the heated fraction only and extracts from two sponge species, both heated and unheated fractions, were active. After dilution only one extract from *Myrmekioderma styx* (#86) showed strong hemagglutinating activity (7.2 μg/mL). The heated fraction lost its activity, suggesting the compound responsible for hemagglutinating activity is probably a lectin or a protein. So far, several compounds with antimicrobial and cytotoxic activities have been isolated from this species [33–36], but no hemagglutinating activity has been reported.

A large number of compounds exhibiting antimicrobial activity with potential biomedical application have been isolated from marine sponges [39]. Therefore, the fact that the majority of the sponges investigated in this study possess antimicrobial activity is not surprising (Suppl. Tables 1a and 1b). Almost all samples (only two samples showed no activity) were able to prevent growth of at least one bacterial strain. Many of the samples inhibited the growth of Gram positive bacteria, but only few affected the growth of Gram negative bacteria. Organic extracts tended to be more active than the aqueous extracts. Diluted samples of those sponges that showed the highest antibacterial activity were retested and results expressed as a minimal inhibitory concentration (MIC, Table 2). The strongest antimicrobial activity was found in the organic extracts from the sponges of the genus *Ircinia* and *Verongula*. All extracts from *I. felix* (#59, #93) showed considerable antimicrobial activity. The most active samples were the butanol extracts from *I. strobilina* (MIC = 30 ng/mL), the acetone extract from *V. gigantea* (#44, MIC = 50 ng/mL) and the butanol extract from *V. rigida* (#38, MIC = 80 ng/mL). The highest antimicrobial activity in the aqueous extracts was found in the unheated extract from *I. felix* (#59). Sponges from the genus *Ircinia* are among the most widely studied in terms of their natural products. A number of different compounds have been isolated and characterized, many of them being antimicrobial [37, 38]. The only extracts that showed considerable activity towards Gram negative strain were those from *Topsentia ophiraphidites* (#99) and *Aplysina archeri* (#61). The antimicrobial activity of the unheated aqueous extract (MIC = 48 μg/mL) was about 100 fold more potent than the heated extracts, again indicating the active compound might be a peptide or a protein. The specificity against Gram negative bacteria is interesting because these bacteria are usually more resistant to antimicrobial compounds due to the lipopolysaccharidic component of their cell wall.

Sepčić *et al.* have screened several aqueous extracts from Mediterranean sponges and found strong anti-AChE activity in one species [40] that was later ascribed to alkylpyridinium polymers [41]. In the present study several extracts showed similar activity, however, after dilution and repeated testing only a few were considered worthy of further investigation (Table 3). Inhibition was generally only moderate, with one notable exception, organic extracts from *Topsentia ophiraphidites* showed considerable inhibitory activity, the most active being the butanolic extract (34 μg) which inhibited almost 50% of AChE activity.

Compounds which inhibit or activate cell protein phosphatases/kinases, such as calyculins, are of special interest because of their role in cell signaling and cell cycle control [42]. Sponge extracts in this study showed both inhibition and activation of PP1 (Suppl. Table 1a and 1b). After dilution of active samples ten sponge extracts from nine species were identified as potentially interesting for further research (Table 4). Several heated aqueous extracts enhanced PP1 activity by up to 2-fold. The only exceptions are the unheated and heated aqueous extracts from *Callyspongia plicifera* (#9) which caused 23% and 44% inhibition, respectively. Organic extracts caused PP1 inhibition only. The most active extracts were those from *I. felix* (#93), *Neofibularia nolitangere* (#83) and from an unidentified sponge (#21). From each of these species at least one organic extract totally inhibited PP1 activity. The concentrations necessary for total enzyme inhibition range from 54 to 790 μg/mL. The interesting PP1 activation by several aqueous heated extracts cannot be explained by the presence of innate protein phosphatases since those should be destroyed by heating. We cannot exclude the possibility that activation is due to the interference between certain compounds in tested extracts with chromophores used in the PP1 inhibition assay.

## 3. Experimental Section

### 3.1. Sponge collection

Sixty-six sponge specimens were studied, represented by 43 sponge species (Table 1a and 1b). Thirty-five were collected by SCUBA by Dr. Daniel Schaft at depths from 5 to 45 m in the reefs of Curaçao (Netherlands Antilles) at several locations: the entrance of Picadera Bay, coastline of Charo and Boca Sami. The specimens were taxonomically determined to species level, seven remained unidentified. The other eight species were collected from Lizard Island (Great Barrier Reef, Queensland, Australia), and identified to at least genus level.

### 3.2. Sample preparation

All sponge samples were lyophilized and dried weight was determined. The total material was divided into two parts; one part for aqueous extraction, the other subjected to extraction with organic solvents. The total mass of freeze-dried sponge samples was within the range from 0.35 g to 36.8 g.

### 3.3. Aqueous extraction

One half of total lyophilized mass of each sponge specimen was homogenized, dissolved in 10 mL of deionized water and extracted for 12 hours with constant shaking (400 rpm at 4 °C) followed by centrifugation (15,000 rpm at 4 °C). Supernatants were removed and divided into two parts. The first part was unheated and was stored in aliquots of 1 mL at −20 °C. The second portion supernatants was boiled for 15 min at 100 °C, cooled and centrifuged for 15 min at 13,000 rpm. The resulting »heated« supernatants were stored in 1 mL aliquots at −20 °C. The dry weight of each sample was determined using 500 μL of each sample which was placed for 30 min into an oven and dried at 120 °C. The dry weight was expressed in mg/mL. Stock concentrations of unheated aqueous extracts were from 5.85 to 95.40 mg/mL (proteins from 0.46 to 44.18 mg/mL), those of heated fractions from 4.80 to 86.72 mg/mL.

### 3.4. Protein determination

The protein content was determined only in unheated samples and measured by BCA protein reagent according to the manufacturers manual (Pierce, USA). Different concentrations of bovine serum albumin (Sigma, USA) were used as a standard. Prior to the addition of the reagent, samples were diluted 1:20 (v/v) with deionized water. The colour formation was determined at 562 nm using microtiter plate reader (Dynex Technologies, USA) after 30 min of incubation.

### 3.5. Extraction with organic solvents

One half of each total lyophilized sponge body mass was macerated and divided into three parts which were placed into three labeled tubes (A, acetone; B, butanol; M, methanol, all solvents were from Merck, Germany). To the each tube, the solvent was added in a way that its volume was about 1 cm above the sample. Tubes were sealed with metal stoppers and parafilm and were shaken overnight at 37 °C. The extracts were filtered and the remaining material was subjected to repeated extraction for 3 hours at 37 °C with constant shaking. Both filtrates were combined and put into Erlenmayer flasks. The solvents were evaporated, and each of the resulting supernatants resuspended in 2 ml of 96% ethanol (Merck, Germany). Dry weight of each sample was determined by drying an aliquot of a sample in a preweighed round bottom flask by evaporation under vacuum at 45 °C. The dry weight was expressed in mg/mL. Stock concentrations were in the following ranges: 0.48 to 39.75 mg/mL for acetone, 1.08 to 72.8 mg/mL for butanol, and 16.84 to 239.11 mg/mL for methanol extracts, respectively.

### 3.6. Hemolytic activity assay

Fresh bovine erythrocytes were used for hemolytic tests. Red blood cells were washed three times in physiological saline prior to use. Finally, they were diluted in a buffer containing 0.13 M NaCl in 0.02 M TRIS-HCl, pH 7.4. The erythrocyte suspension had an initial absorption value of 1.0 ± 0.01 AU at 650 nm. Hemolytic activity was assayed using a microplate UV/VIS absorbtion reader (Dynex, USA). To each well 100 μL of buffer was added followed by 20 μL ethanol-dissolved acetone, buthanol or methanol extracts, respectively. Finally, 100 μL of erythrocyte suspension was added to initiate the assay. Time course of hemolysis was monitored until the absorption dropped to the half of its initial value (approx. 0.250 AU) and expressed as a half-time of hemolysis (t_50_). All measurements were done at 25 °C. Blind experiments were conducted, using 20 μL water or absolute ethanol as controls. Samples showing the highest hemolytic activity were further diluted (1:2, 1:10 and 1:20 for organic extracts and 1:10, 1:100 and 1:1000 for aqueous extracts, respectively). Assays with diluted samples were repeated accordingly.

### 3.7. Antibacterial activity assay

Antibacterial activity was tested by means of a standard agar plate diffusion assay. The Gram positive *Bacillus subtilis* and the Gram negative *Escherichia coli* bacterial strains (obtained from the microbial collection at the Chair for Microbiology, Biotechnical Faculty, University of Ljubljana, Slovenia) were used. Precultured bacteria (grown in Luria broth media, Sigma, USA) were used for the inoculation of Luria broth agar plates in a final concentration of 5 × 10^5^ cells/L. Four holes (1 cm in diameter) were made into each agar plate and filled with 100 μL of unheated or heated samples in the case of aqueous extracts. In the case of organic extracts to the each hole 100 μL of ethanol-dissolved acetone, butanol or methanol samples were added. The fourth hole was used for the control and was filled with ethanol. The inhibition zone for each sample was determined after the overnight incubation of plates at 37°C. Samples showing the highest inhibition of bacterial growth were further diluted with deionized water or ethanol (1:10, 1: 100 and 1:1000), tests were repeated and minimal inhibitory concentrations were calculated (MIC = the concentration in μg/mL that inhibits the growth of tested microorganism 1 mm from the rim of the hole).

### 3.8. Hemagglutination assay

Samples obtained by aqueous extraction were tested for hemagglutinating activity. Fresh bovine erythrocytes were washed twice in buffer as described above (see hemolytic activity assay). Two per cent final erythrocyte suspension was prepared using the same buffer. The erythrocyte suspension (100 μL) was added to each well of a 96 round-well microtitre plate, followed by 25 μL of samples. Hemagglutination was visually inspected after 45 min of incubation at room temperature.

### 3.9. Acetylcholinesterase inhibition assay

The acetylcholinesterase assay was performed according to the method of Ellman *et al.* [28]. Briefly, AChE from electric eel (Sigma, USA), was dissolved in 100 mM phosphate buffer (pH 7.3) to achieve 500 EU/mL. Prior to the test, enzyme was 100 fold diluted in the same buffer. To each microtiter plate well 140 μL of the Ellman reagent (5,5-dithiobis-2-nitrobenzoic acid) in 25 mM phosphate buffer (pH 7.0), 10 μL of acetylcholine (ACh) in 1 mM final concentration, 20 ul of sponge sample (aqueous or organic), and finally 50 μL of AChE was added to start the reaction. Deionized (20 μL) water or ethanol (20 μL) was used as controls. The time course of the enzymatic reaction was monitored for 12 min at 412 nm and 25 °C. Samples that showed significant AChE inhibitory activity were further diluted (1:10 and 1:100) and tested accordingly. UV/VIS microplate reader (Dynex, USA) was used in all assays.

### 3.10. Protein phosphatase 1 inhibition/activation assay

The effects of sponge samples on PP1 activity were monitored colorimetrically according to Tubaro *et al.* [29] using a microplate reader (Dynex, USA). Rabbit recombinant α-isoform PP1 expressed in *E. coli* (Sigma, USA) was the enzyme used. To each well of the microtiter plate 150 μL of buffer (40 mM TRIS/HCl, 34 mM MgCl_2_.6H_2_O, 4 mM EDTA and 4 mM DL-DTT, pH 8.4), 50 μL of the substrate (141 mM *p*-nitrophenil phosphate) and 2 μL of sponge samples (2 μL of deionized water or ethanol in the controls) was added. The reaction was started by the addition of 50 μL of buffer-dissolved PP1 (0.25 U/mL). Samples that showed activity were further diluted (1:10 and 1:100 v/v). All reactions were monitored for 12 min at 25 °C for the colour development at 405 nm.

## 4. Conclusions

We conclude that almost all sponge extracts tested in this study showed at least one activity, but only few represent a promising source for further research into their active components. Most of these components are probably smaller organic compounds whose activity is not destroyed by heating. However, some active components might be larger, most probably proteins, whose activity is lost upon heating as a result of denaturation. The most interesting species in terms of different activities were *Ircinia felix*, *Topsentia ophiraphidites* and *Pandaros acanthifolium*. Bioassay guided isolation and characterization of the active components from these species may yield useful candidates in the search for new pharmaceutical leads.

## Supplementary Data

Supplement Table 1a List of species from Curaçao (Netherland Antilles).

**Table:** 

Species	S #	Hemolytic activity	Antibacterial activity (inhibition zone mm)		Hem- agglutination	AChE inhibition					PP1 activation/inhibition				

			*B. subtilis*	*E. coli*		aqueous		Organic			aqueous		organic		
					
						aq	aqh	A	B	M	aq	aqh	A	B	M
*Agelas clathrodes*	81	+++ ^A,B,M^	2 ^aq^, 3 aqh					+++	+	+++					i
			2 ^A^, 2 ^B^, 5 ^M^												
*Agelas conifera*	34		.5 ^A^, 8 ^B^, 10 ^M^	.5 ^A^, 8 ^B^, 10 ^M^		**+++**	**+++**		**+++**	**+++**					
*Agelas conifera*	97		4 ^A^, 2 ^B^, 9 ^M^	1.5 ^A^,1 ^B^, 6 ^M^		**+++**	**+++**	**+**		**+++**		i	i	i	
*Agelas dispar*	58									+++					iii
*Agelas dispar*	88		2 ^A^, 2 ^B^, 3 ^M^				+	+	++	++		i	i	ii	i
*Agelas schmidti*	54		9 ^A^, 9 ^B^, 9 ^M^	0.5 ^B^											
*Aplysina archeri*	40		3.5 ^aq^ 3 ^A^, 5 ^B^, 4 ^M^	2 ^A^, 1 ^B^, 1 ^M^	+ ^aq^	+++	+++	+	+	++			iii	iii	iii
*Aplysina archeri*	61	++ ^A,B^	6 ^aq^ 3 ^A^, 3 ^B^, 6 ^M^	2 ^aq^ 1 ^A^, 1 ^B^, 3 ^M^	+ aqh	+				+++		ii			
*Alpysina cauliformis*	84	++ ^A,B^, + ^M^	2 ^aq^ 2 ^A^,2 ^B^,3 ^M^							++	ii	ii			
*Aplysina fistularis*	57	++ ^A,B^	2 ^A^, 2 ^B^, 2 ^M^	1 ^A^, 1 ^B^, .5 ^M^								ii			
*Aplysina fulva*	10	+++ ^A^, ++ ^B,M^	6 ^aq^ 3 ^A^, 3 ^B^, 10 ^M^	4 ^aq^ 2 ^A^, 1 ^B^, 6 ^M^		+	+			+++	ii	ii			
*Aplysina lacunosa*	41	++ ^A,B^, + ^M^	2 ^A^, 2 ^B^, 2 ^M^				+			+					
*Aplysina lacunosa*	112		3 ^A^, 3 ^B^, 2 ^M^		+ ^aq^	+++	+++	+	+	+++			ii	ii	
*Callyspongia plicifera*	9		2 ^A^, 1 ^B^, 2 ^M^							+	i	i	ii	ii	i
*Callyspongia plicifera*	67		3 ^A^, 2 ^B^, 2 ^M^							+		ii	i	ii	i
*Callyspongia plicifera*	103		3 ^A^, 2 ^B^, 1 ^M^	1 ^A^, 2 ^B^, 1 ^M^									i	ii	iii
*Callyspongia plicifera*	127		2 ^A^, 2 ^B^, 1 ^M^									a			
*Callyspongia vaginalis*	15		1 ^A^, 1 ^B^	1 ^B^									ii	i	iii
*Callyspongia vaginalis*	48		2 ^A^, 2 ^B^, 2 ^M^	1 ^B^, 2 ^M^						+		ii	ii	iii	iii
*Callyspongia vaginalis*	66		3 ^A^, 4 ^B^, 1 ^M^		+ ^aq^			+	+	++		i	i	i	i
*Geodia neptuni*	87	+ ^A,M^	3 ^A^, 2 ^B^, 2 ^M^					++				ii			
*Holopsamma helwigi*	5		3 ^A^, 5 ^B^, 2 ^M^		+ ^aq^					+			ii	ii	ii
*Holopsamma helwigi*	122				+ ^aq^										
*Iotrochota birotulata*	77		4 ^A^, 4 ^B^, 4 ^M^	1 ^A^, 4 ^B^, 4 ^M^	+ ^aq^, + aqh	++	+	+++	++	+++			i	iii	
*Ircinia campana*	70	+ ^A,B^	7 ^A^, 5 ^B^, 6 ^M^					+	+	++		a	ii	i	i
*Ircinia felix*	59	+++ ^A,B,M^	5 ^aq^, 5.3 aqh 9 ^A^,10 ^B^, 9 ^M^				+	++	++	++		a	i	ii	ii
*Ircinia felix*	93	+ ^A,M^, ++ ^B^	3 ^aq^, 2.1 aqh 13 ^A^,12 ^B^, 9 ^M^			++	+	+	++	++		a	i	ii	iii
*Ircinia felix*	129	+++ ^A^	5 ^A^, 2 ^B^, 5 ^M^										ii	i	ii
*Ircinia strobilina*	56	++ ^A^, + ^B^	0.5 ^aq^ 10 ^A^, 3 ^B^, 4 ^M^					+		+		a	i	i	i
*Ircinia strobilina*	124	+++ ^A,M^, ++ ^B^	9 ^A^, 2 ^B^, 10 ^M^							+	ii				i
*Lissodendoryx colombiensis*	51	+ ^aq^ ++ ^A^, + ^B^	4 ^aq^ 2 ^A^, 2 ^B^, 7 ^M^	2 ^aq^ 2 ^M^	+ ^aq^						ii	ii	i	i	
*Lissodendoryx colombiensis*	110	+++ ^A^	10 ^A^, 3 ^B^, 4 ^M^	1 ^A^				++	+	+++			i		ii
*Myrmekioderma styx*	86	++ ^A^, + ^B^	4 ^A^, 4 ^B^, 3 ^M^	.5 ^A^, .5 ^B^, .5 ^M^	+ ^aq^, + aqh			++	++			a			
*Neofibularia nolitangere*	49	++ ^A^, + ^B^	2 ^A^, 2 ^B^, 2 ^M^	.5 ^A^											
*Neofibularia nolitangere*	83		3 ^A^, 3 ^B^, 2 ^M^	1 ^M^					+	+			ii	iii	iii
*Neofibularia nolitangere*	94		4 ^A^, 4 ^B^, 3 ^M^	2 ^A^, 1 ^M^		++						i	i	i	i
*Pandaros acanthifolium*	14		3 ^A^, 2 ^B^, 2 ^M^	1 ^A^, 1 ^B^, 1 ^M^										i	ii
*Pandaros acanthifolium*	76	+++ ^aq^	3 ^A^, 2 ^B^, 5 ^M^	.5 ^A^, .5 ^B^, .5 ^M^				+	+	+	iii	iii	ii	i	i
*Pseudoceratina crassa*	2		3 ^aq^ 7 ^A^, 3 ^B^, 7 ^M^	1 ^B^	+ ^aq^	+	+	+	+	++				i	ii
*Pseudoceratina crassa*	104	+++ ^A^, ++ ^M^	3 ^A^, 2 ^B^, 2 ^M^		+ ^aq^	+		++			ii	ii	i	i	
*Scopalina ruetzleri*	78		2 ^A^, 1 ^B^, 2 ^M^			+	+						i		i
*Spheciospongia vesparium*	45		2 ^A^, 2 ^B^, 2 ^M^	1 ^A^,1 ^M^			+			++			i	i	
*Spheciospongia vesparium*	69	+++ ^aq^ ++ ^B^, +++ ^M^	2 ^A^, 2 ^M^			+	+			++	iii	iii		i	iii
*Topsentia ophiraphidites*	99	+++ ^aq^, +++ aqh, +++ ^A^, ++ ^M^	7 ^aq^, 8 aqh 7 ^A^, 7 ^B^, 7 ^M^	3 aqh 4 ^A^, 4 ^B^, 4 ^M^		+++	++	+++	+++	+++	ii	ii	ii	i	
*Tridideum misolidum*	79		3 ^A^, 2 ^B^, 2 ^M^	1 ^A^, 2 ^B^									ii	ii	ii
*Verongula gigantea*	44		5 ^A^, 4 ^B^, 4 ^M^	1 ^A^, 1 ^B^, 1 ^M^		+++	++	+	+	++		a	i	i	i
*Verongula rigida*	38		5 ^aq^, 5.2 aqh	1 aqh				+	++	++		a	i	i	i
			6 ^A^, 6 ^B^, 6 ^M^	1 ^A^, 1 ^B^											
*Verongula rigida*	105	+ ^A^	5 ^aq^ 7 ^A^, 3 ^B^, 10 ^M^	1 ^A^, 2 ^M^		+		+		+					
*Xestospongia muta*	53	+++ ^B,M^	10 ^A^, 7 ^B^, 6 ^M^												i
*Xestospongia muta*	95		4 ^A^, 6 ^B^, 5 ^M^						+	+		i			
Unidentified A21	114	+ ^aq^	2 ^A^, 1 ^B^, 5 ^M^	2 ^M^											
Unidentified A33	28	++ ^A,B,M^	3 ^aq^, 2 aqh		+ ^aq^	+++	+++			+++		ii		iii	iii
Unidentified A34	25	++ ^A,B^	5 ^A^, 4 ^B^, 4 ^M^												
Unidentified 1	21		2 ^A^, 2 ^B^, 3 ^M^	1 ^A^, 1 ^B^		++	++	+	+	+++		ii	iii	iii	iii
Unidentified 2	32	+ ^B^	4 ^A^, 4 ^B^, 3 ^M^	1 ^A^, 1 ^B^, 1 ^M^		+++	+++	+	++	++			ii	ii	ii
Unidentified 3	96		2 ^A^, 3 ^B^, 2 ^M^						+	++			i	i	ii
Unidentified 4	117	++ ^aq^	3 ^A^, 3 ^B^, 2 ^M^		+ ^aq^	+	+	+	+	+		a	i	i	i

aq aqueous unheated,

aqh aqueous heated

Organic extracts: A (acetone), B (butanol), M (methanol)

Hemolytic activity: +, moderate activity (t_50_ between 10 and 15 min); ++, strong activity (t_50_ between 5 and 10 min); +++, very strong activity (t_50_ between 0 and 5 min).

t_50_ = half-time of hemolysis, e.g. the time in which 50% of erythrocytes are lysed.

AChE inhibition: +, moderate inhibition (0–33%); ++, strong inhibition (34–66%); +++, very strong inhibition (67–100%).

PP1 activation/inhibition: a, activation (up to 100%); i, moderate inhibition (0–33%); ii, strong inhibition (34–66%); iii, very strong inhibition (67–100%).

S# sample number.

Supplement Table 1b List of species from Lizard island (Great Barrier Reef, Queensland, Australia).

**Table:** 

Species	S.#	Hemolytic activity	Antibacterial activity (inhibition zone mm)		Hem- aglutination	AChE inhibition					PP1 activation/inhibition				

			*B. subtilis*	*E. coli*		aqueous		Organic			aqueous		organic		
					
						aq	aqh	A	B	M	aq	aqh	A	B	M
*Hyrtios erecta*	LI-10	+ ^A^	4 ^A^, 4 ^B^, 4 ^M^												iii
*Ircinia sp.*	LI-39		2 ^A^, 2 ^B^, 2 ^M^			++									
*Ircinia sp.*	107	++ ^A,B^, + ^M^	9 ^A^,10 ^B^, 9 ^M^					+	+	+		i	ii	ii	i
*Ircinia cf. Abseits*	132	+ ^A^, ++ ^B^	8 ^A^, 8 ^B^, 6 ^M^					+	+	+		a	i	ii	i
*Pericharax heteroraphis*	LI-35	+++ ^A^, ++ ^B^, + ^M^	2 ^A^, 2 ^B^, 2 ^M^									a			i
*Phakellia stipitata*	LI-5		4 ^A^, 3 ^B^, 4 ^M^												ii
*Spongia sp.*	LI-43	+ ^A^	2 ^A^, 2 ^B^, 2 ^M^			+									i
*Thorectandra sp.*	LI-27	+ ^A^, + ^B^	3 ^A^, 2 ^B^, 3 ^M^		+ ^aq^	+		+++	++	+++			i		i
*Xestospongia pacifica*	LI-47		3 ^A^, 3 ^B^, 3 ^M^	2 ^A^, 1 ^M^		+++	+++	++	+++	+++	iii	i	iii	iii	iii

aq aqueous unheated,

aqh aqueous heated.

Organic extracts: A (acetone), B (butanol), M (methanol).

Hemolytic activity: +, moderate activity (t_50_ between 10 and 15 min); ++, strong activity (t_50_ between 5 and 10 min); +++, very strong activity (t_50_ between 0 and 5 min).

t_50_ = half-time of hemolysis, e.g. the time in which 50% of erythrocytes are lysed.

AChE inhibition: +, moderate inhibition (0–33%); ++, strong inhibition (34–66%); +++, very strong inhibition (67–100%).

PP1 activation/inhibition: a, activation (up to 100%); i, moderate inhibition (0–33%); ii, strong inhibition (34–66%); iii, very strong inhibition (67–100%).

S# sample number.

## Figures and Tables

**Table 1:** Hemolytic activity of the most active sponge extracts.

		Aqueous extracts				Organic extracts^1^					
		
Sponge species	S#	Amount in the assay (μg/mL)		Hemolytic activity		Amount in the assay (μg/mL)			Hemolytic activity		

		Unheated	Heated	Unheated	Heated	A	B	M	A	B	M
*Agelas clathrodes*	81							32			+++
*Ircinia felix*	59					2.6	43	88	+++	+++	+++
*Lissodendoryx colombiensis*	110					25			+++		
*Pandaros acanthifolium*	76	60.5/10.4*		+++							
*Spheciospongia vesparium*	69	408/143*		+++							
*Topsentia ophiraphidites*	99	2.5/0.22*	2.5/0*	+++	+++	157			+++		

1 organic extracts: A (acetone), B (butanol), M (methanol).

+++, hemolytic activity (t_50_ between 0 and 5 min). t_50_ = half-time of hemolysis, e.g. the time in which 50% of erythrocytes are lysed.

* protein.

S# sample number.

**Table 2:** Antibacterial activity presented as minimal inhibitory concentration (MIC).

Sponge species	Sample #	*B. subtilis* (MIC μg/mL)					*E. coli* (MIC μg/mL)				

		Aqueous extracts		Organic extracts			Aqueous extracts		Organic extracts^1^		

		unheated	heated	A	B	M	unheated	heated	A	B	M
*Agelas conifera*	34				2					2	
*Agelas conifera*	97					3					
*Agelas schmidti*	54			1							
*Aplysina archeri*	40	3050		36	8	27					
*Aplysina archeri*	61						50				
*Callyspongia vaginalis*	66				4						
*Callyspongia plicifera*	103									120	
*Holopsamma helwigi*	5				0.4						
*Hyrtios erecta*	LI-10			5							
*Ircinia campana*	70			0. 7	7	3					
*Ircinia felix*	59	80	2750	0. 1	2	0. 4					
*Ircinia felix*	93	2.5		0. 7	1	32					
*Ircinia* sp.	107			0. 9	0.9	0. 1					
*Ircinia cf. abseits*	132			0. 3	0. 7	2					
*Ircinia strobilina*	56			0. 1	0.03						
*Ircinia strobilina*	124			17							
*Lissodendoryx colombiensis*	51					70					
*Neofibularia nolitangere*	94				2						
*Pseudoceratina crassa*	2			0. 4							
*Topsentia ophiraphidites*	99			34			50	5420	3470		
*Verongula gigantea*	44			0.05	0. 4						
*Verongula rigida*	38	1650	1310	0. 6	0. 08	1.2					
*Verongula rigida*	105	1660				53					
*Xestospongia muta*	53			1.8							
Unidentified 2				6	6						
Unidentified A34	25			9							

1 Organic extracts: A (acetone), B (butanol), M (methanol).

**Table 3:** Anti-acetylcholinesterase (AChE) activity of the most active sponge extracts.

		Aqueous extracts				Organic extracts 1					
		
Sponge species	S#	Amount in the assay (μg/mL)		AChE inhibition (%)		Amount in the assay (μg/mL)			AChE inhibition (%)		

		Unheated	heated	Unheated	Heated	A	B	M	A	B	M
*Agelas clathrodes*	81	337/140*	337/0*	23	23						
*Agelas conifera*	97							30			28
*Topsentia ophiraphidites*	99					31	34	217	31	46	62
*Xestospongia pacifica*	LI-47	500/116*	492/0*	31	31		181	532		31	31
Unidentified A33	28		0.78/0*					76			23

1 Organic extracts: A (acetone), B (butanol), M (methanol).

* protein;

S# sample number.

**Table 4:** Modulation of protein phosphatase 1 (PP1) activity.

Species	S#	Amount μg/mL					PP1 *activation*/inhibition				

		aqueous		organic			aqueous		organic		
		
		unheated	heate	A	B	M	unheated	heated	A	B	M
			d								
*Callyspongia plicifera*	9	58	48				23	44			
*Ircinia felix*	59	283/78*	273					*x2*			
*Ircinia felix*	93	244/70*	200	176	275	790		*x1.5*	33	67	100
*Ircinia strobilina*	56	192/55*	177					*x1.4*			
*Ircinia* cf. *abseits*	13	346/41*	227					*x1.5*			
	2										
*Neofibularia nolitangere*	83			28	54	167			67	100	100
*Spheciospongia*	69					78					50
*vesparium*											
*Verongula gigantea*	44	388/138*	319					*x2*			
*Verongula rigida*	38	164/54*	130					*x2*			
Unidentified 1	21			73	117	673			100	100	100

Organic extracts: A (acetone), B (butanol), M (methanol).

Activation is indicated in italics, and denotes a factor by which the PP1 activity is enhanced.

Inhibition is indicated in normal text, and denotes the % of inhibition.

* protein.

S# sample number.
